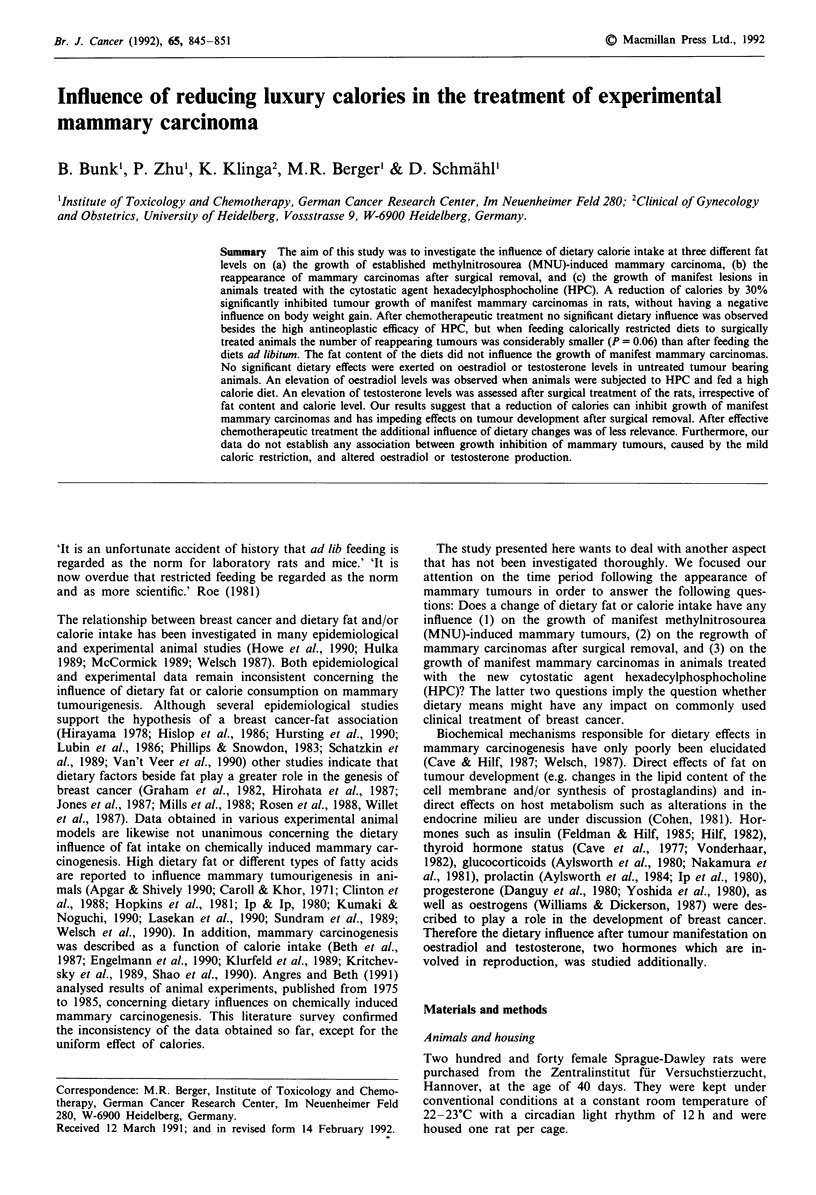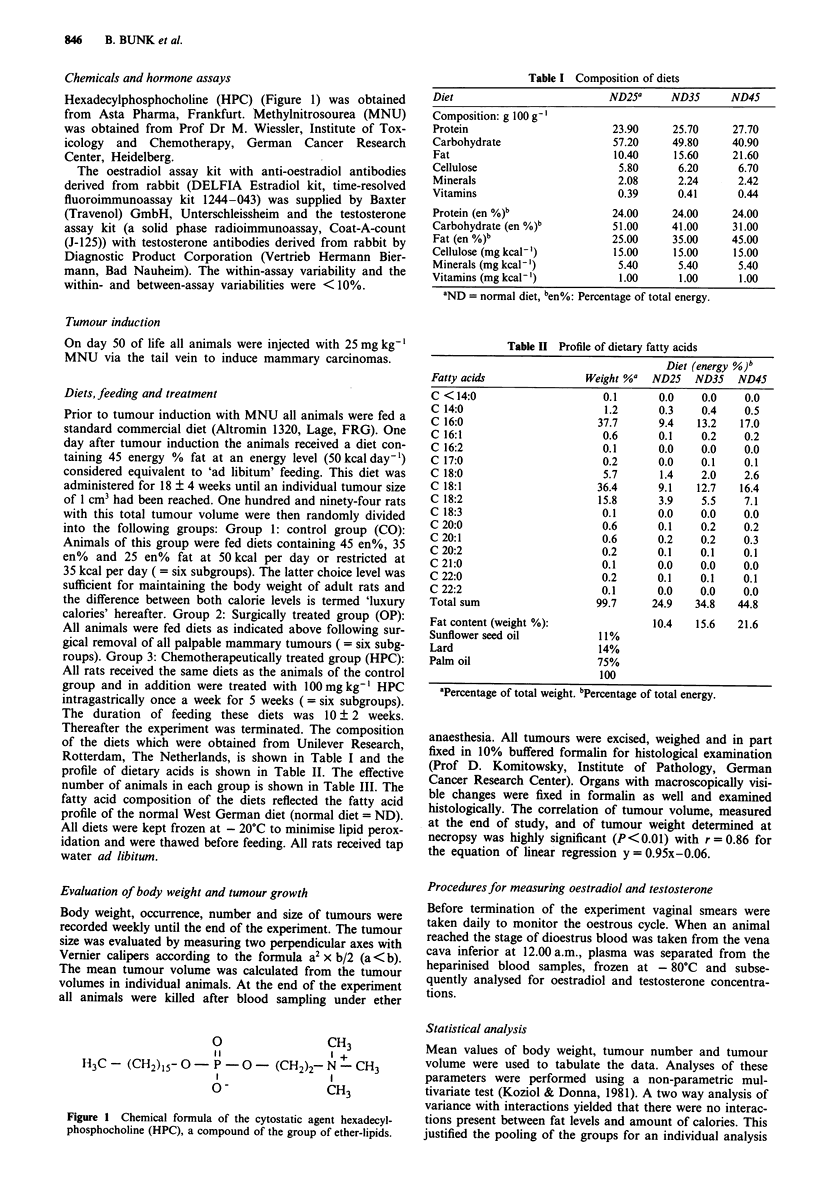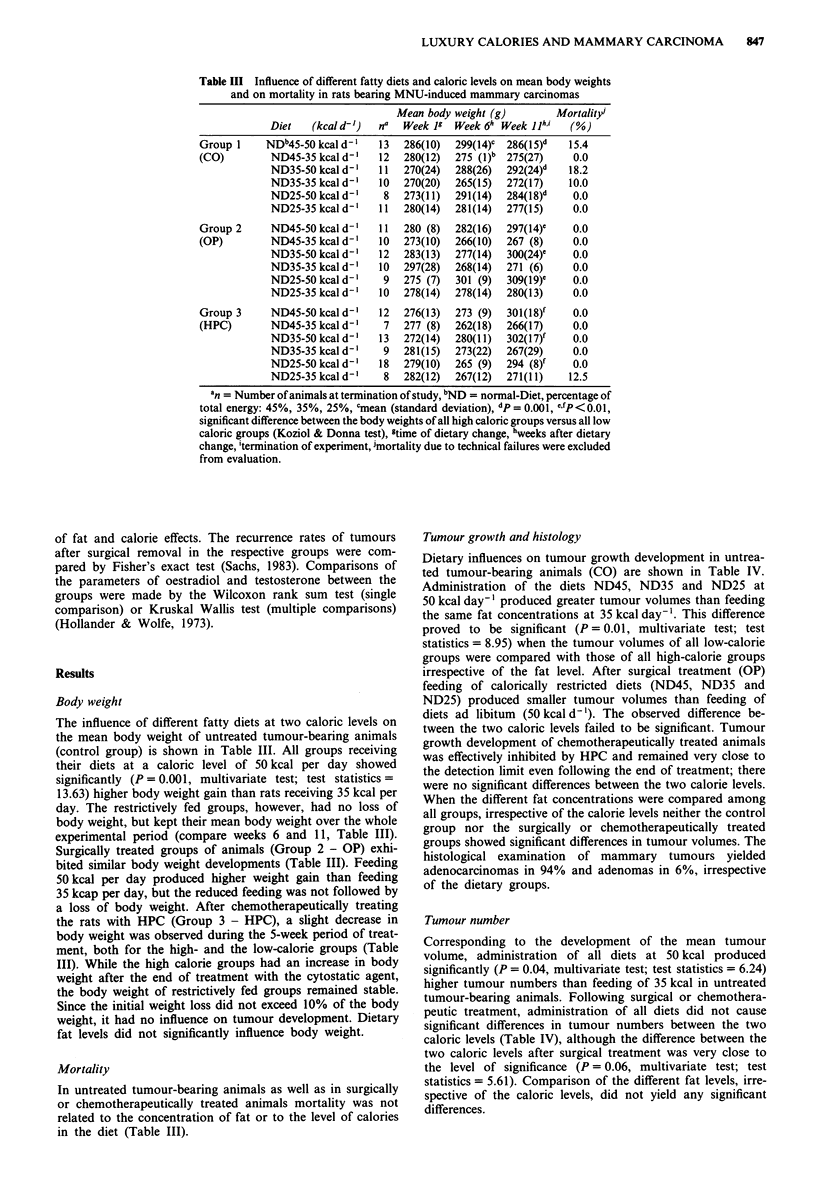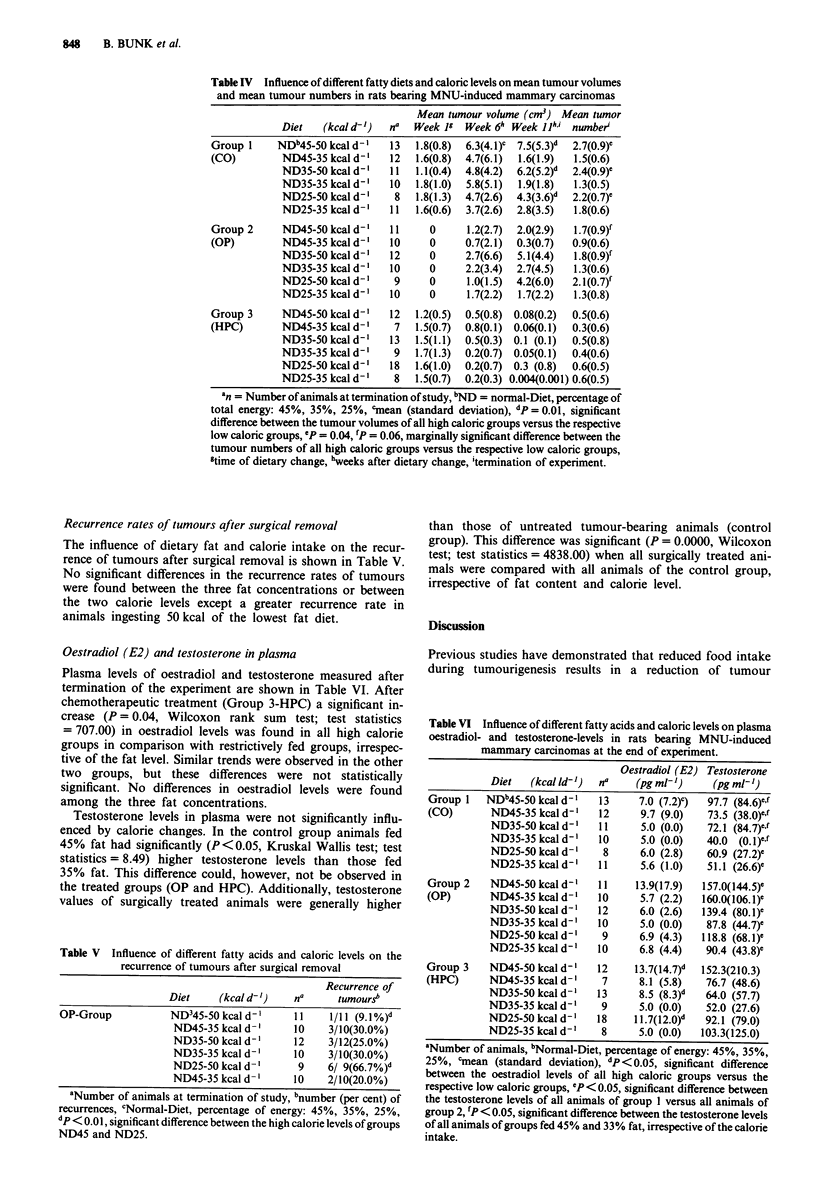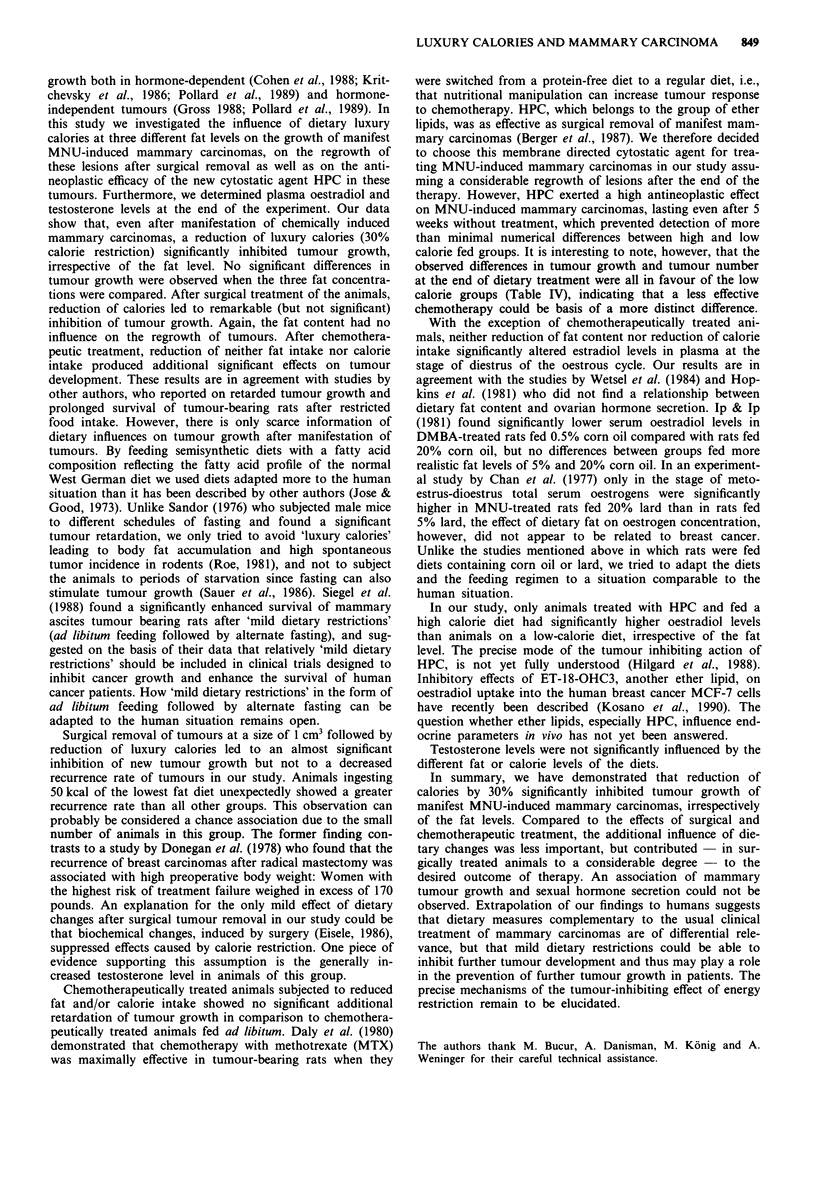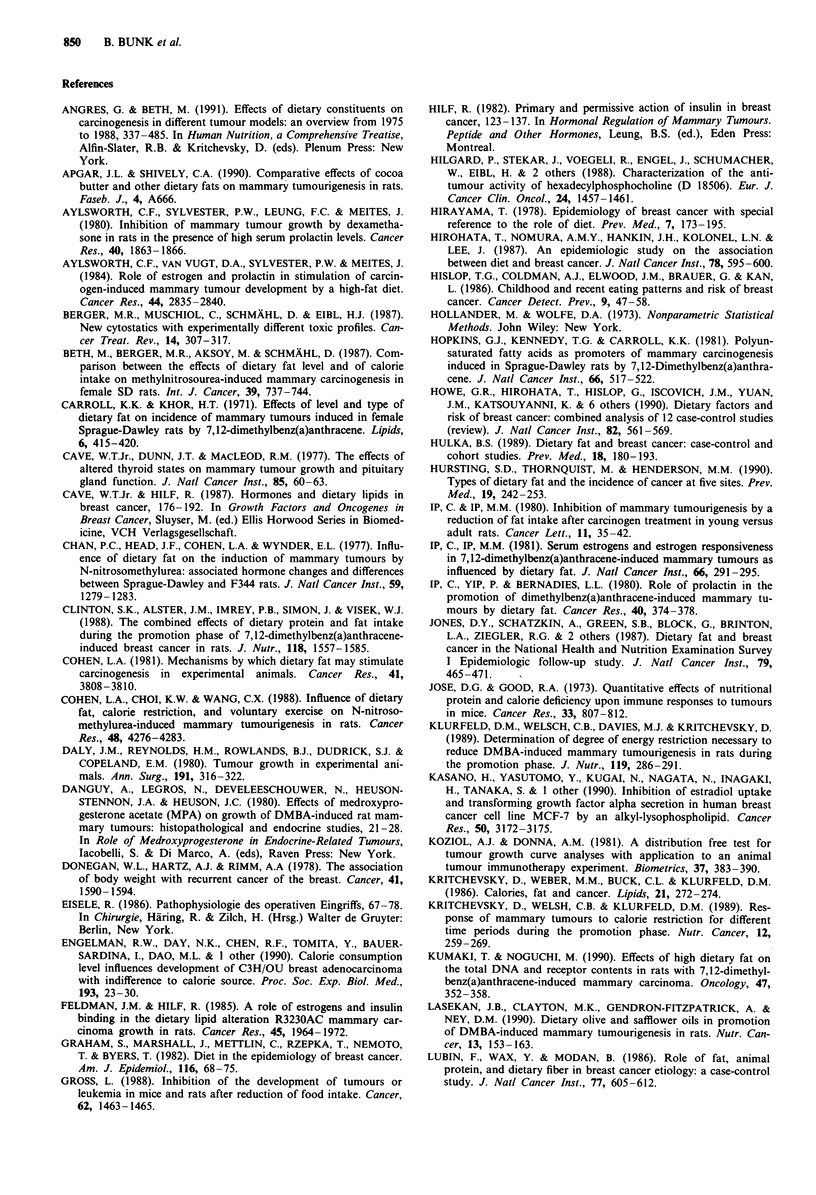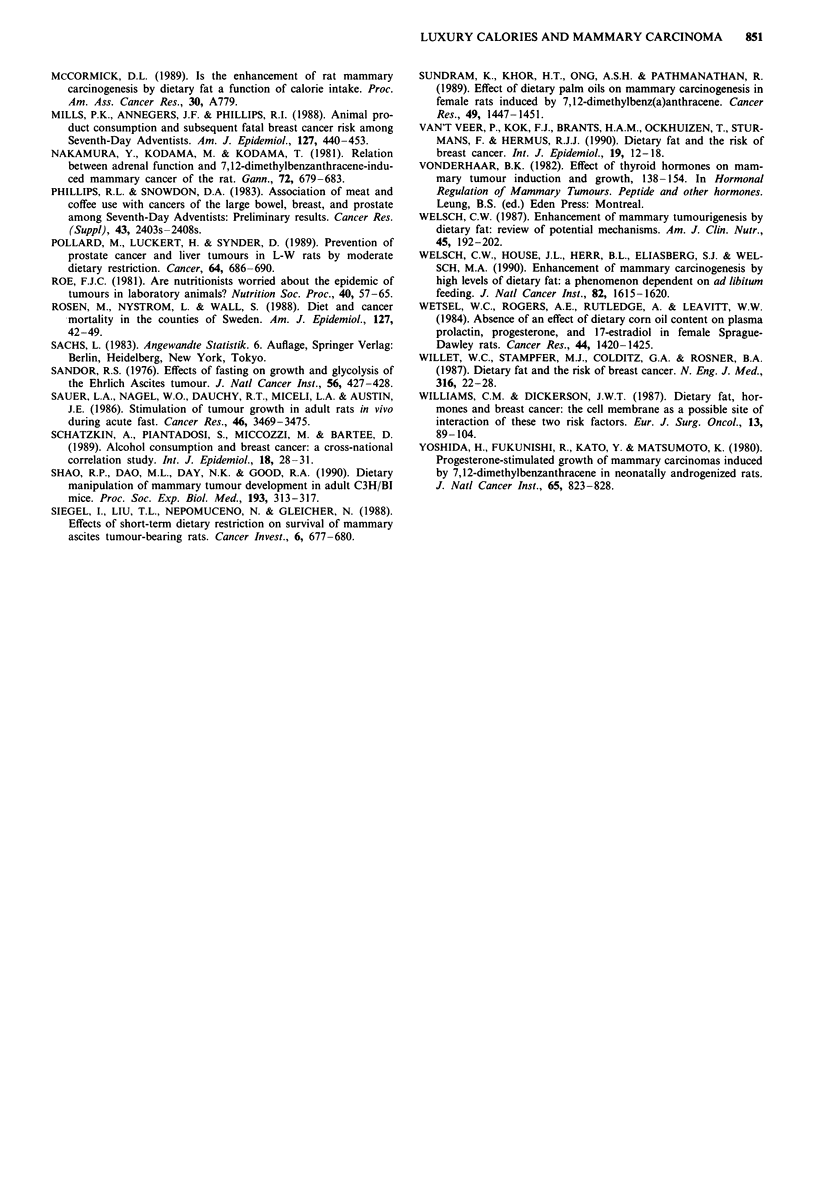# Influence of reducing luxury calories in the treatment of experimental mammary carcinoma.

**DOI:** 10.1038/bjc.1992.179

**Published:** 1992-06

**Authors:** B. Bunk, P. Zhu, K. Klinga, M. R. Berger, D. Schmähl

**Affiliations:** Institute of Toxicology and Chemotherapy, German Cancer Research Center, Heidelberg.

## Abstract

The aim of this study was to investigate the influence of dietary calorie intake at three different fat levels on (a) the growth of established methylnitrosourea (MNU)-induced mammary carcinoma, (b) the reappearance of mammary carcinomas after surgical removal, and (c) the growth of manifest lesions in animals treated with the cytostatic agent hexadecylphosphocholine (HPC). A reduction of calories by 30% significantly inhibited tumour growth of manifest mammary carcinomas in rats, without having a negative influence on body weight gain. After chemotherapeutic treatment no significant dietary influence was observed besides the high antineoplastic efficacy of HPC, but when feeding calorically restricted diets to surgically treated animals the number of reappearing tumours was considerably smaller (P = 0.06) than after feeding the diets ad libitum. The fat content of the diets did not influence the growth of manifest mammary carcinomas. No significant dietary effects were exerted on oestradiol or testosterone levels in untreated tumour bearing animals. An elevation of oestradiol levels was observed when animals were subjected to HPC and fed a high calorie diet. An elevation of testosterone levels was assessed after surgical treatment of the rats, irrespective of fat content and calorie level. Our results suggest that a reduction of calories can inhibit growth of manifest mammary carcinomas and has impeding effects on tumour development after surgical removal. After effective chemotherapeutic treatment the additional influence of dietary changes was of less relevance. Furthermore, our data do not establish any association between growth inhibition of mammary tumours, caused by the mild caloric restriction, and altered oestradiol or testosterone production.


Br. J. Cancer (1992), 65, 845-851 © Macmillan Press Ltd., 1992

### Influence of reducing luxury calories in the treatment of experimental mammary carcinoma

B. Bunk[1], P. Zhu[1], K. Klinga[2], M.R. Berger[1] & D. Schmähl[1]

[1]*Institute of Toxicology and Chemotherapy, German Cancer Research Center, Im Neuenheimer Feld 280;* [2]*Clinical of Gynecology and Obstetrics, University of Heidelberg, Vossstrasse 9, W-6900 Heidelberg, Germany.*

**Summary** The aim of this study was to investigate the influence of dietary calorie intake at three different fat levels on (a) the growth of established methylnitrosourea (MNU)-induced mammary carcinoma, (b) the reappearance of mammary carcinomas after surgical removal, and (c) the growth of manifest lesions in animals treated with the cytostatic agent hexadecylphosphocholine (HPC). A reduction of calories by 30% significantly inhibited tumour growth of manifest mammary carcinomas in rats, without having a negative influence on body weight gain. After chemotherapeutic treatment no significant dietary influence was observed besides the high antineoplastic efficacy of HPC, but when feeding calorically restricted diets to surgically treated animals the number of reappearing tumours was considerably smaller ($P = 0.06$) than after feeding the diets *ad libitum*. The fat content of the diets did not influence the growth of manifest mammary carcinomas. No significant dietary effects were exerted on oestradiol or testosterone levels in untreated tumour bearing animals. An elevation of oestradiol levels was observed when animals were subjected to HPC and fed a high calorie diet. An elevation of testosterone levels was assessed after surgical treatment of the rats, irrespective of fat content and calorie level. Our results suggest that a reduction of calories can inhibit growth of manifest mammary carcinomas and has impeding effects on tumour development after surgical removal. After effective chemotherapeutic treatment the additional influence of dietary changes was of less relevance. Furthermore, our data do not establish any association between growth inhibition of mammary tumours, caused by the mild caloric restriction, and altered oestradiol or testosterone production.

'It is an unfortunate accident of history that *ad lib* feeding is regarded as the norm for laboratory rats and mice.' 'It is now overdue that restricted feeding be regarded as the norm and as more scientific.' Roe (1981)

The relationship between breast cancer and dietary fat and/or calorie intake has been investigated in many epidemiological and experimental animal studies (Howe *et al.*, 1990; Hulka 1989; McCormick 1989; Welsch 1987). Both epidemiological and experimental data remain inconsistent concerning the influence of dietary fat or calorie consumption on mammary tumourigenesis. Although several epidemiological studies support the hypothesis of a breast cancer-fat association (Hirayama 1978; Hislop *et al.*, 1986; Hursting *et al.*, 1990; Lubin *et al.*, 1986; Phillips & Snowdon, 1983; Schatzkin *et al.*, 1989; Van't Veer *et al.*, 1990) other studies indicate that dietary factors beside fat play a greater role in the genesis of breast cancer (Graham *et al.*, 1982, Hirohata *et al.*, 1987; Jones *et al.*, 1987; Mills *et al.*, 1988; Rosen *et al.*, 1988, Willet *et al.*, 1987). Data obtained in various experimental animal models are likewise not unanimous concerning the dietary influence of fat intake on chemically induced mammary carcinogenesis. High dietary fat or different types of fatty acids are reported to influence mammary tumourigenesis in animals (Apgar & Shively 1990; Caroll & Khor, 1971; Clinton *et al.*, 1988; Hopkins *et al.*, 1981; Ip & Ip, 1980; Kumaki & Noguchi, 1990; Lasekan *et al.*, 1990; Sundram *et al.*, 1989; Welsch *et al.*, 1990). In addition, mammary carcinogenesis was described as a function of calorie intake (Beth *et al.*, 1987; Engelmann *et al.*, 1990; Klurfeld *et al.*, 1989; Kritchevsky *et al.*, 1989, Shao *et al.*, 1990). Angres and Beth (1991) analysed results of animal experiments, published from 1975 to 1985, concerning dietary influences on chemically induced mammary carcinogenesis. This literature survey confirmed the inconsistency of the data obtained so far, except for the uniform effect of calories.

Correspondence: M.R. Berger, Institute of Toxicology and Chemotherapy, German Cancer Research Center, Im Neuenheimer Feld 280, W-6900 Heidelberg, Germany.
Received 12 March 1991; and in revised form 14 February 1992.

The study presented here wants to deal with another aspect that has not been investigated thoroughly. We focused our attention on the time period following the appearance of mammary tumours in order to answer the following questions: Does a change of dietary fat or calorie intake have any influence (1) on the growth of manifest methylnitrosourea (MNU)-induced mammary tumours, (2) on the regrowth of mammary carcinomas after surgical removal, and (3) on the growth of manifest mammary carcinomas in animals treated with the new cytostatic agent hexadecylphosphocholine (HPC)? The latter two questions imply the question whether dietary means might have any impact on commonly used clinical treatment of breast cancer.

Biochemical mechanisms responsible for dietary effects in mammary carcinogenesis have only poorly been elucidated (Cave & Hilf, 1987; Welsch, 1987). Direct effects of fat on tumour development (e.g. changes in the lipid content of the cell membrane and/or synthesis of prostaglandins) and indirect effects on host metabolism such as alterations in the endocrine milieu are under discussion (Cohen, 1981). Hormones such as insulin (Feldman & Hilf, 1985; Hilf, 1982), thyroid hormone status (Cave *et al.*, 1977; Vonderhaar, 1982), glucocorticoids (Aylsworth *et al.*, 1980; Nakamura *et al.*, 1981), prolactin (Aylsworth *et al.*, 1984; Ip *et al.*, 1980), progesterone (Danguy *et al.*, 1980; Yoshida *et al.*, 1980), as well as oestrogens (Williams & Dickerson, 1987) were described to play a role in the development of breast cancer. Therefore the dietary influence after tumour manifestation on oestradiol and testosterone, two hormones which are involved in reproduction, was studied additionally.

### Materials and methods

### *Animals and housing*

Two hundred and forty female Sprague-Dawley rats were purchased from the Zentralinstitut für Versuchstierzucht, Hannover, at the age of 40 days. They were kept under conventional conditions at a constant room temperature of 22-23°C with a circadian light rhythm of 12 h and were housed one rat per cage.

### *Chemicals and hormone assays*

Hexadecylphosphocholine (HPC) (Figure 1) was obtained from Asta Pharma, Frankfurt. Methylnitrosourea (MNU) was obtained from Prof Dr M. Wiessler, Institute of Toxicology and Chemotherapy, German Cancer Research Center, Heidelberg.

The oestradiol assay kit with anti-oestradiol antibodies derived from rabbit (DELFIA Estradiol kit, time-resolved fluoroimmunoassay kit 1244-043) was supplied by Baxter (Travenol) GmbH, Unterschleissheim and the testosterone assay kit (a solid phase radioimmunoassay, Coat-A-count (J-125)) with testosterone antibodies derived from rabbit by Diagnostic Product Corporation (Vertrieb Hermann Biermann, Bad Nauheim). The within-assay variability and the within- and between-assay variabilities were <10%.

### *Tumour induction*

On day 50 of life all animals were injected with $25\,mg\,kg^{-1}$ MNU via the tail vein to induce mammary carcinomas.

### *Diets, feeding and treatment*

Prior to tumour induction with MNU all animals were fed a standard commercial diet (Altromin 1320, Lage, FRG). One day after tumour induction the animals received a diet containing 45 energy % fat at an energy level ($50\,kcal\,day^{-1}$) considered equivalent to 'ad libitum' feeding. This diet was administered for 18 ± 4 weeks until an individual tumour size of 1 $cm^3$ had been reached. One hundred and ninety-four rats with this total tumour volume were then randomly divided into the following groups: Group 1: control group (CO): Animals of this group were fed diets containing 45 en%, 35 en% and 25 en% fat at 50 kcal per day or restricted at 35 kcal per day ( = six subgroups). The latter choice level was sufficient for maintaining the body weight of adult rats and the difference between both calorie levels is termed 'luxury calories' hereafter. Group 2: Surgically treated group (OP): All animals were fed diets as indicated above following surgical removal of all palpable mammary tumours ( = six subgroups). Group 3: Chemotherapeutically treated group (HPC): All rats received the same diets as the animals of the control group and in addition were treated with $100\,mg\,kg^{-1}$ HPC intragastrically once a week for 5 weeks ( = six subgroups). The duration of feeding these diets was 10 ± 2 weeks. Thereafter the experiment was terminated. The composition of the diets which were obtained from Unilever Research, Rotterdam, The Netherlands, is shown in Table I and the profile of dietary acids is shown in Table II. The effective number of animals in each group is shown in Table III. The fatty acid composition of the diets reflected the fatty acid profile of the normal West German diet (normal diet = ND). All diets were kept frozen at − 20°C to minimise lipid peroxidation and were thawed before feeding. All rats received tap water *ad libitum*.

### *Evaluation of body weight and tumour growth*

Body weight, occurrence, number and size of tumours were recorded weekly until the end of the experiment. The tumour size was evaluated by measuring two perpendicular axes with Vernier calipers according to the formula $a^2 \times b/2$ (a<b). The mean tumour volume was calculated from the tumour volumes in individual animals. At the end of the experiment all animals were killed after blood sampling under ether anaesthesia. All tumours were excised, weighed and in part fixed in 10% buffered formalin for histological examination (Prof D. Komitowsky, Institute of Pathology, German Cancer Research Center). Organs with macroscopically visible changes were fixed in formalin as well and examined histologically. The correlation of tumour volume, measured at the end of study, and of tumour weight determined at necropsy was highly significant ($P<0.01$) with $r = 0.86$ for the equation of linear regression $y = 0.95x - 0.06$.

**Figure 1** Chemical formula of the cytostatic agent hexadecylphosphocholine (HPC), a compound of the group of ether-lipids.

**Table I** Composition of diets

| *Diet* | *ND25*[a] | *ND35* | *ND45* |
|---|---|---|---|
| Composition: $g\,100\,g^{-1}$ | | | |
| Protein | 23.90 | 25.70 | 27.70 |
| Carbohydrate | 57.20 | 49.80 | 40.90 |
| Fat | 10.40 | 15.60 | 21.60 |
| Cellulose | 5.80 | 6.20 | 6.70 |
| Minerals | 2.08 | 2.24 | 2.42 |
| Vitamins | 0.39 | 0.41 | 0.44 |
| Protein (en %)[b] | 24.00 | 24.00 | 24.00 |
| Carbohydrate (en %)[b] | 51.00 | 41.00 | 31.00 |
| Fat (en %)[b] | 25.00 | 35.00 | 45.00 |
| Cellulose ($mg\,kcal^{-1}$) | 15.00 | 15.00 | 15.00 |
| Minerals ($mg\,kcal^{-1}$) | 5.40 | 5.40 | 5.40 |
| Vitamins ($mg\,kcal^{-1}$) | 1.00 | 1.00 | 1.00 |

[a]ND = normal diet, [b]en%: Percentage of total energy.

**Table II** Profile of dietary fatty acids

| *Fatty acids* | *Weight %*[a] | *Diet (energy %)*[b] *ND25* | *ND35* | *ND45* |
|---|---|---|---|---|
| C <14:0 | 0.1 | 0.0 | 0.0 | 0.0 |
| C 14:0 | 1.2 | 0.3 | 0.4 | 0.5 |
| C 16:0 | 37.7 | 9.4 | 13.2 | 17.0 |
| C 16:1 | 0.6 | 0.1 | 0.2 | 0.2 |
| C 16:2 | 0.1 | 0.0 | 0.0 | 0.0 |
| C 17:0 | 0.2 | 0.0 | 0.1 | 0.1 |
| C 18:0 | 5.7 | 1.4 | 2.0 | 2.6 |
| C 18:1 | 36.4 | 9.1 | 12.7 | 16.4 |
| C 18:2 | 15.8 | 3.9 | 5.5 | 7.1 |
| C 18:3 | 0.1 | 0.0 | 0.0 | 0.0 |
| C 20:0 | 0.6 | 0.1 | 0.2 | 0.2 |
| C 20:1 | 0.6 | 0.2 | 0.2 | 0.3 |
| C 20:2 | 0.2 | 0.1 | 0.1 | 0.1 |
| C 21:0 | 0.1 | 0.0 | 0.0 | 0.0 |
| C 22:0 | 0.2 | 0.1 | 0.1 | 0.1 |
| C 22:2 | 0.1 | 0.0 | 0.0 | 0.0 |
| Total sum | 99.7 | 24.9 | 34.8 | 44.8 |
| Fat content (weight %): | | 10.4 | 15.6 | 21.6 |
| Sunflower seed oil | 11% | | | |
| Lard | 14% | | | |
| Palm oil | 75% | | | |
| | 100 | | | |

[a]Percentage of total weight. [b]Percentage of total energy.

### *Procedures for measuring oestradiol and testosterone*

Before termination of the experiment vaginal smears were taken daily to monitor the oestrous cycle. When an animal reached the stage of dioestrus blood was taken from the vena cava inferior at 12.00 a.m., plasma was separated from the heparinised blood samples, frozen at − 80°C and subsequently analysed for oestradiol and testosterone concentrations.

### *Statistical analysis*

Mean values of body weight, tumour number and tumour volume were used to tabulate the data. Analyses of these parameters were performed using a non-parametric multivariate test (Koziol & Donna, 1981). A two way analysis of variance with interactions yielded that there were no interactions present between fat levels and amount of calories. This justified the pooling of the groups for an individual analysis

**Table III** Influence of different fatty diets and caloric levels on mean body weights and on mortality in rats bearing MNU-induced mammary carcinomas

| | | | *Mean body weight (g)* | | | *Mortality*[j] |
|---|---|---|---|---|---|---|
| | *Diet (kcal d*$^{-1}$*)* | *n*[a] | *Week 1*[g] | *Week 6*[h] | *Week 11*[h,i] | *(%)* |
| Group 1 (CO) | ND[b]45-50 kcal d$^{-1}$ | 13 | 286(10) | 299(14)[c] | 286(15)[d] | 15.4 |
| | ND45-35 kcal d$^{-1}$ | 12 | 280(12) | 275 (1)[b] | 275(27) | 0.0 |
| | ND35-50 kcal d$^{-1}$ | 11 | 270(24) | 288(26) | 292(24)[d] | 18.2 |
| | ND35-35 kcal d$^{-1}$ | 10 | 270(20) | 265(15) | 272(17) | 10.0 |
| | ND25-50 kcal d$^{-1}$ | 8 | 273(11) | 291(14) | 284(18)[d] | 0.0 |
| | ND25-35 kcal d$^{-1}$ | 11 | 280(14) | 281(14) | 277(15) | 0.0 |
| Group 2 (OP) | ND45-50 kcal d$^{-1}$ | 11 | 280 (8) | 282(16) | 297(14)[e] | 0.0 |
| | ND45-35 kcal d$^{-1}$ | 10 | 273(10) | 266(10) | 267 (8) | 0.0 |
| | ND35-50 kcal d$^{-1}$ | 12 | 283(13) | 277(14) | 300(24)[e] | 0.0 |
| | ND35-35 kcal d$^{-1}$ | 10 | 297(28) | 268(14) | 271 (6) | 0.0 |
| | ND25-50 kcal d$^{-1}$ | 9 | 275 (7) | 301 (9) | 309(19)[e] | 0.0 |
| | ND25-35 kcal d$^{-1}$ | 10 | 278(14) | 278(14) | 280(13) | 0.0 |
| Group 3 (HPC) | ND45-50 kcal d$^{-1}$ | 12 | 276(13) | 273 (9) | 301(18)[f] | 0.0 |
| | ND45-35 kcal d$^{-1}$ | 7 | 277 (8) | 262(18) | 266(17) | 0.0 |
| | ND35-50 kcal d$^{-1}$ | 13 | 272(14) | 280(11) | 302(17)[f] | 0.0 |
| | ND35-35 kcal d$^{-1}$ | 9 | 281(15) | 273(22) | 267(29) | 0.0 |
| | ND25-50 kcal d$^{-1}$ | 18 | 279(10) | 265 (9) | 294 (8)[f] | 0.0 |
| | ND25-35 kcal d$^{-1}$ | 8 | 282(12) | 267(12) | 271(11) | 12.5 |

[a]*n* = Number of animals at termination of study, [b]ND = normal-Diet, percentage of total energy: 45%, 35%, 25%, [c]mean (standard deviation), [d]$P = 0.001$, [e,f]$P < 0.01$, significant difference between the body weights of all high caloric groups versus all low caloric groups (Koziol & Donna test), [g]time of dietary change, [h]weeks after dietary change, [i]termination of experiment, [j]mortality due to technical failures were excluded from evaluation.

of fat and calorie effects. The recurrence rates of tumours after surgical removal in the respective groups were compared by Fisher's exact test (Sachs, 1983). Comparisons of the parameters of oestradiol and testosterone between the groups were made by the Wilcoxon rank sum test (single comparison) or Kruskal Wallis test (multiple comparisons) (Hollander & Wolfe, 1973).

### Results

### *Body weight*

The influence of different fatty diets at two caloric levels on the mean body weight of untreated tumour-bearing animals (control group) is shown in Table III. All groups receiving their diets at a caloric level of 50 kcal per day showed significantly ($P = 0.001$, multivariate test; test statistics = 13.63) higher body weight gain than rats receiving 35 kcal per day. The restrictively fed groups, however, had no loss of body weight, but kept their mean body weight over the whole experimental period (compare weeks 6 and 11, Table III). Surgically treated groups of animals (Group 2 – OP) exhibited similar body weight developments (Table III). Feeding 50 kcal per day produced higher weight gain than feeding 35 kcap per day, but the reduced feeding was not followed by a loss of body weight. After chemotherapeutically treating the rats with HPC (Group 3 – HPC), a slight decrease in body weight was observed during the 5-week period of treatment, both for the high- and the low-calorie groups (Table III). While the high calorie groups had an increase in body weight after the end of treatment with the cytostatic agent, the body weight of restrictively fed groups remained stable. Since the initial weight loss did not exceed 10% of the body weight, it had no influence on tumour development. Dietary fat levels did not significantly influence body weight.

### *Mortality*

In untreated tumour-bearing animals as well as in surgically or chemotherapeutically treated animals mortality was not related to the concentration of fat or to the level of calories in the diet (Table III).

### *Tumour growth and histology*

Dietary influences on tumour growth development in untreated tumour-bearing animals (CO) are shown in Table IV. Administration of the diets ND45, ND35 and ND25 at 50 kcal day$^{-1}$ produced greater tumour volumes than feeding the same fat concentrations at 35 kcal day$^{-1}$. This difference proved to be significant ($P = 0.01$, multivariate test; test statistics = 8.95) when the tumour volumes of all low-calorie groups were compared with those of all high-calorie groups irrespective of the fat level. After surgical treatment (OP) feeding of calorically restricted diets (ND45, ND35 and ND25) produced smaller tumour volumes than feeding of diets ad libitum (50 kcal d$^{-1}$). The observed difference between the two caloric levels failed to be significant. Tumour growth development of chemotherapeutically treated animals was effectively inhibited by HPC and remained very close to the detection limit even following the end of treatment; there were no significant differences between the two calorie levels. When the different fat concentrations were compared among all groups, irrespective of the calorie levels neither the control group nor the surgically or chemotherapeutically treated groups showed significant differences in tumour volumes. The histological examination of mammary tumours yielded adenocarcinomas in 94% and adenomas in 6%, irrespective of the dietary groups.

### *Tumour number*

Corresponding to the development of the mean tumour volume, administration of all diets at 50 kcal produced significantly ($P = 0.04$, multivariate test; test statistics = 6.24) higher tumour numbers than feeding of 35 kcal in untreated tumour-bearing animals. Following surgical or chemotherapeutic treatment, administration of all diets did not cause significant differences in tumour numbers between the two caloric levels (Table IV), although the difference between the two caloric levels after surgical treatment was very close to the level of significance ($P = 0.06$, multivariate test; test statistics = 5.61). Comparison of the different fat levels, irrespective of the caloric levels, did not yield any significant differences.

**Table IV** Influence of different fatty diets and caloric levels on mean tumour volumes and mean tumour numbers in rats bearing MNU-induced mammary carcinomas

| | Diet (kcal $d^{-1}$) | $n^a$ | Mean tumour volume ($cm^3$) Week 1[g] | Week 6[h] | Week 11[h,i] | Mean tumor number[i] |
|---|---|---|---|---|---|---|
| Group 1 | ND[b]45-50 kcal $d^{-1}$ | 13 | 1.8(0.8) | 6.3(4.1)[c] | 7.5(5.3)[d] | 2.7(0.9)[e] |
| (CO) | ND45-35 kcal $d^{-1}$ | 12 | 1.6(0.8) | 4.7(6.1) | 1.6(1.9) | 1.5(0.6) |
| | ND35-50 kcal $d^{-1}$ | 11 | 1.1(0.4) | 4.8(4.2) | 6.2(5.2)[d] | 2.4(0.9)[e] |
| | ND35-35 kcal $d^{-1}$ | 10 | 1.8(1.0) | 5.8(5.1) | 1.9(1.8) | 1.3(0.5) |
| | ND25-50 kcal $d^{-1}$ | 8 | 1.8(1.3) | 4.7(2.6) | 4.3(3.6)[d] | 2.2(0.7)[e] |
| | ND25-35 kcal $d^{-1}$ | 11 | 1.6(0.6) | 3.7(2.6) | 2.8(3.5) | 1.8(0.6) |
| Group 2 | ND45-50 kcal $d^{-1}$ | 11 | 0 | 1.2(2.7) | 2.0(2.9) | 1.7(0.9)[f] |
| (OP) | ND45-35 kcal $d^{-1}$ | 10 | 0 | 0.7(2.1) | 0.3(0.7) | 0.9(0.6) |
| | ND35-50 kcal $d^{-1}$ | 12 | 0 | 2.7(6.6) | 5.1(4.4) | 1.8(0.9)[f] |
| | ND35-35 kcal $d^{-1}$ | 10 | 0 | 2.2(3.4) | 2.7(4.5) | 1.3(0.6) |
| | ND25-50 kcal $d^{-1}$ | 9 | 0 | 1.0(1.5) | 4.2(6.0) | 2.1(0.7)[f] |
| | ND25-35 kcal $d^{-1}$ | 10 | 0 | 1.7(2.2) | 1.7(2.2) | 1.3(0.8) |
| Group 3 | ND45-50 kcal $d^{-1}$ | 12 | 1.2(0.5) | 0.5(0.8) | 0.08(0.2) | 0.5(0.6) |
| (HPC) | ND45-35 kcal $d^{-1}$ | 7 | 1.5(0.7) | 0.8(0.1) | 0.06(0.1) | 0.3(0.6) |
| | ND35-50 kcal $d^{-1}$ | 13 | 1.5(1.1) | 0.5(0.3) | 0.1 (0.1) | 0.5(0.8) |
| | ND35-35 kcal $d^{-1}$ | 9 | 1.7(1.3) | 0.2(0.7) | 0.05(0.1) | 0.4(0.6) |
| | ND25-50 kcal $d^{-1}$ | 18 | 1.6(1.0) | 0.2(0.7) | 0.3 (0.8) | 0.6(0.5) |
| | ND25-35 kcal $d^{-1}$ | 8 | 1.5(0.7) | 0.2(0.3) | 0.004(0.001) | 0.6(0.5) |

[a]$n$ = Number of animals at termination of study, [b]ND = normal-Diet, percentage of total energy: 45%, 35%, 25%, [c]mean (standard deviation), [d]$P = 0.01$, significant difference between the tumour volumes of all high caloric groups versus the respective low caloric groups, [e]$P = 0.04$, [f]$P = 0.06$, marginally significant difference between the tumour numbers of all high caloric groups versus the respective low caloric groups, [g]time of dietary change, [h]weeks after dietary change, [i]termination of experiment.

*Recurrence rates of tumours after surgical removal*

The influence of dietary fat and calorie intake on the recurrence of tumours after surgical removal is shown in Table V. No significant differences in the recurrence rates of tumours were found between the three fat concentrations or between the two calorie levels except a greater recurrence rate in animals ingesting 50 kcal of the lowest fat diet.

*Oestradiol (E2) and testosterone in plasma*

Plasma levels of oestradiol and testosterone measured after termination of the experiment are shown in Table VI. After chemotherapeutic treatment (Group 3-HPC) a significant increase ($P = 0.04$, Wilcoxon rank sum test; test statistics = 707.00) in oestradiol levels was found in all high calorie groups in comparison with restrictively fed groups, irrespective of the fat level. Similar trends were observed in the other two groups, but these differences were not statistically significant. No differences in oestradiol levels were found among the three fat concentrations.

Testosterone levels in plasma were not significantly influenced by calorie changes. In the control group animals fed 45% fat had significantly ($P < 0.05$, Kruskal Wallis test; test statistics = 8.49) higher testosterone levels than those fed 35% fat. This difference could, however, not be observed in the treated groups (OP and HPC). Additionally, testosterone values of surgically treated animals were generally higher than those of untreated tumour-bearing animals (control group). This difference was significant ($P = 0.0000$, Wilcoxon test; test statistics = 4838.00) when all surgically treated animals were compared with all animals of the control group, irrespective of fat content and calorie level.

**Table V** Influence of different fatty acids and caloric levels on the recurrence of tumours after surgical removal

| | Diet (kcal $d^{-1}$) | $n^a$ | Recurrence of tumours[b] |
|---|---|---|---|
| OP-Group | ND[c]45-50 kcal $d^{-1}$ | 11 | 1/11 (9.1%)[d] |
| | ND45-35 kcal $d^{-1}$ | 10 | 3/10(30.0%) |
| | ND35-50 kcal $d^{-1}$ | 12 | 3/12(25.0%) |
| | ND35-35 kcal $d^{-1}$ | 10 | 3/10(30.0%) |
| | ND25-50 kcal $d^{-1}$ | 9 | 6/ 9(66.7%)[d] |
| | ND45-35 kcal $d^{-1}$ | 10 | 2/10(20.0%) |

[a]Number of animals at termination of study, [b]number (per cent) of recurrences, [c]Normal-Diet, percentage of energy: 45%, 35%, 25%, [d]$P < 0.01$, significant difference between the high calorie levels of groups ND45 and ND25.

### Discussion

Previous studies have demonstrated that reduced food intake during tumourigenesis results in a reduction of tumour

**Table VI** Influence of different fatty acids and caloric levels on plasma oestradiol- and testosterone-levels in rats bearing MNU-induced mammary carcinomas at the end of experiment.

| | Diet (kcal $ld^{-1}$) | $n^a$ | Oestradiol (E2) (pg $ml^{-1}$) | Testosterone (pg $ml^{-1}$) |
|---|---|---|---|---|
| Group 1 | ND[b]45-50 kcal $d^{-1}$ | 13 | 7.0 (7.2)[c]) | 97.7 (84.6)[e,f] |
| (CO) | ND45-35 kcal $d^{-1}$ | 12 | 9.7 (9.0) | 73.5 (38.0)[e,f] |
| | ND35-50 kcal $d^{-1}$ | 11 | 5.0 (0.0) | 72.1 (84.7)[e,f] |
| | ND35-35 kcal $d^{-1}$ | 10 | 5.0 (0.0) | 40.0 (0.1)[e,f] |
| | ND25-50 kcal $d^{-1}$ | 8 | 6.0 (2.8) | 60.9 (27.2)[e] |
| | ND25-35 kcal $d^{-1}$ | 11 | 5.6 (1.0) | 51.1 (26.6)[e] |
| Group 2 | ND45-50 kcal $d^{-1}$ | 11 | 13.9(17.9) | 157.0(144.5)[e] |
| (OP) | ND45-35 kcal $d^{-1}$ | 10 | 5.7 (2.2) | 160.0(106.1)[e] |
| | ND35-50 kcal $d^{-1}$ | 12 | 6.0 (2.6) | 139.4 (80.1)[e] |
| | ND35-35 kcal $d^{-1}$ | 10 | 5.0 (0.0) | 87.8 (44.7)[e] |
| | ND25-50 kcal $d^{-1}$ | 9 | 6.9 (4.3) | 118.8 (68.1)[e] |
| | ND25-35 kcal $d^{-1}$ | 10 | 6.8 (4.4) | 90.4 (43.8)[e] |
| Group 3 | ND45-50 kcal $d^{-1}$ | 12 | 13.7(14.7)[d] | 152.3(210.3) |
| (HPC) | ND45-35 kcal $d^{-1}$ | 7 | 8.1 (5.8) | 76.7 (48.6) |
| | ND35-50 kcal $d^{-1}$ | 13 | 8.5 (8.3)[d] | 64.0 (57.7) |
| | ND35-35 kcal $d^{-1}$ | 9 | 5.0 (0.0) | 52.0 (27.6) |
| | ND25-50 kcal $d^{-1}$ | 18 | 11.7(12.0)[d] | 92.1 (79.0) |
| | ND25-35 kcal $d^{-1}$ | 8 | 5.0 (0.0) | 103.3(125.0) |

[a]Number of animals, [b]Normal-Diet, percentage of energy: 45%, 35%, 25%, [c]mean (standard deviation), [d]$P < 0.05$, significant difference between the oestradiol levels of all high caloric groups versus the respective low caloric groups, [e]$P < 0.05$, significant difference between the testosterone levels of all animals of group 1 versus all animals of group 2, [f]$P < 0.05$, significant difference between the testosterone levels of all animals of groups fed 45% and 33% fat, irrespective of the calorie intake.

growth both in hormone-dependent (Cohen *et al.*, 1988; Kritchevsky *et al.*, 1986; Pollard *et al.*, 1989) and hormone-independent tumours (Gross 1988; Pollard *et al.*, 1989). In this study we investigated the influence of dietary luxury calories at three different fat levels on the growth of manifest MNU-induced mammary carcinomas, on the regrowth of these lesions after surgical removal as well as on the anti-neoplastic efficacy of the new cytostatic agent HPC in these tumours. Furthermore, we determined plasma oestradiol and testosterone levels at the end of the experiment. Our data show that, even after manifestation of chemically induced mammary carcinomas, a reduction of luxury calories (30% calorie restriction) significantly inhibited tumour growth, irrespective of the fat level. No significant differences in tumour growth were observed when the three fat concentrations were compared. After surgical treatment of the animals, reduction of calories led to remarkable (but not significant) inhibition of tumour growth. Again, the fat content had no influence on the regrowth of tumours. After chemotherapeutic treatment, reduction of neither fat intake nor calorie intake produced additional significant effects on tumour development. These results are in agreement with studies by other authors, who reported on retarded tumour growth and prolonged survival of tumour-bearing rats after restricted food intake. However, there is only scarce information of dietary influences on tumour growth after manifestation of tumours. By feeding semisynthetic diets with a fatty acid composition reflecting the fatty acid profile of the normal West German diet we used diets adapted more to the human situation than it has been described by other authors (Jose & Good, 1973). Unlike Sandor (1976) who subjected male mice to different schedules of fasting and found a significant tumour retardation, we only tried to avoid 'luxury calories' leading to body fat accumulation and high spontaneous tumor incidence in rodents (Roe, 1981), and not to subject the animals to periods of starvation since fasting can also stimulate tumour growth (Sauer *et al.*, 1986). Siegel *et al.* (1988) found a significantly enhanced survival of mammary ascites tumour bearing rats after 'mild dietary restrictions' (*ad libitum* feeding followed by alternate fasting), and suggested on the basis of their data that relatively 'mild dietary restrictions' should be included in clinical trials designed to inhibit cancer growth and enhance the survival of human cancer patients. How 'mild dietary restrictions' in the form of *ad libitum* feeding followed by alternate fasting can be adapted to the human situation remains open.

Surgical removal of tumours at a size of 1 $cm^3$ followed by reduction of luxury calories led to an almost significant inhibition of new tumour growth but not to a decreased recurrence rate of tumours in our study. Animals ingesting 50 kcal of the lowest fat diet unexpectedly showed a greater recurrence rate than all other groups. This observation can probably be considered a chance association due to the small number of animals in this group. The former finding contrasts to a study by Donegan *et al.* (1978) who found that the recurrence of breast carcinomas after radical mastectomy was associated with high preoperative body weight: Women with the highest risk of treatment failure weighed in excess of 170 pounds. An explanation for the only mild effect of dietary changes after surgical tumour removal in our study could be that biochemical changes, induced by surgery (Eisele, 1986), suppressed effects caused by calorie restriction. One piece of evidence supporting this assumption is the generally increased testosterone level in animals of this group.

Chemotherapeutically treated animals subjected to reduced fat and/or calorie intake showed no significant additional retardation of tumour growth in comparison to chemotherapeutically treated animals fed *ad libitum*. Daly *et al.* (1980) demonstrated that chemotherapy with methotrexate (MTX) was maximally effective in tumour-bearing rats when they were switched from a protein-free diet to a regular diet, i.e., that nutritional manipulation can increase tumour response to chemotherapy. HPC, which belongs to the group of ether lipids, was as effective as surgical removal of manifest mammary carcinomas (Berger *et al.*, 1987). We therefore decided to choose this membrane directed cytostatic agent for treating MNU-induced mammary carcinomas in our study assuming a considerable regrowth of lesions after the end of the therapy. However, HPC exerted a high antineoplastic effect on MNU-induced mammary carcinomas, lasting even after 5 weeks without treatment, which prevented detection of more than minimal numerical differences between high and low calorie fed groups. It is interesting to note, however, that the observed differences in tumour growth and tumour number at the end of dietary treatment were all in favour of the low calorie groups (Table IV), indicating that a less effective chemotherapy could be basis of a more distinct difference.

With the exception of chemotherapeutically treated animals, neither reduction of fat content nor reduction of calorie intake significantly altered estradiol levels in plasma at the stage of diestrus of the oestrous cycle. Our results are in agreement with the studies by Wetsel *et al.* (1984) and Hopkins *et al.* (1981) who did not find a relationship between dietary fat content and ovarian hormone secretion. Ip & Ip (1981) found significantly lower serum oestradiol levels in DMBA-treated rats fed 0.5% corn oil compared with rats fed 20% corn oil, but no differences between groups fed more realistic fat levels of 5% and 20% corn oil. In an experimental study by Chan *et al.* (1977) only in the stage of metoestrus-dioestrus total serum oestrogens were significantly higher in MNU-treated rats fed 20% lard than in rats fed 5% lard, the effect of dietary fat on oestrogen concentration, however, did not appear to be related to breast cancer. Unlike the studies mentioned above in which rats were fed diets containing corn oil or lard, we tried to adapt the diets and the feeding regimen to a situation comparable to the human situation.

In our study, only animals treated with HPC and fed a high calorie diet had significantly higher oestradiol levels than animals on a low-calorie diet, irrespective of the fat level. The precise mode of the tumour inhibiting action of HPC, is not yet fully understood (Hilgard *et al.*, 1988). Inhibitory effects of ET-18-OHC3, another ether lipid, on oestradiol uptake into the human breast cancer MCF-7 cells have recently been described (Kosano *et al.*, 1990). The question whether ether lipids, especially HPC, influence endocrine parameters *in vivo* has not yet been answered.

Testosterone levels were not significantly influenced by the different fat or calorie levels of the diets.

In summary, we have demonstrated that reduction of calories by 30% significantly inhibited tumour growth of manifest MNU-induced mammary carcinomas, irrespectively of the fat levels. Compared to the effects of surgical and chemotherapeutic treatment, the additional influence of dietary changes was less important, but contributed — in surgically treated animals to a considerable degree — to the desired outcome of therapy. An association of mammary tumour growth and sexual hormone secretion could not be observed. Extrapolation of our findings to humans suggests that dietary measures complementary to the usual clinical treatment of mammary carcinomas are of differential relevance, but that mild dietary restrictions could be able to inhibit further tumour development and thus may play a role in the prevention of further tumour growth in patients. The precise mechanisms of the tumour-inhibiting effect of energy restriction remain to be elucidated.

The authors thank M. Bucur, A. Danisman, M. König and A. Weninger for their careful technical assistance.

### References

ANGRES, G. & BETH, M. (1991). Effects of dietary constituents on carcinogenesis in different tumour models: an overview from 1975 to 1988, 337–485. In *Human Nutrition, a Comprehensive Treatise*, Alfin-Slater, R.B. & Kritchevsky, D. (eds). Plenum Press: New York.

APGAR, J.L. & SHIVELY, C.A. (1990). Comparative effects of cocoa butter and other dietary fats on mammary tumourigenesis in rats. *Faseb. J.*, **4**, A666.

AYLSWORTH, C.F., SYLVESTER, P.W., LEUNG, F.C. & MEITES, J. (1980). Inhibition of mammary tumour growth by dexamethasone in rats in the presence of high serum prolactin levels. *Cancer Res.*, **40**, 1863–1866.

AYLSWORTH, C.F., VAN VUGT, D.A., SYLVESTER, P.W. & MEITES, J. (1984). Role of estrogen and prolactin in stimulation of carcinogen-induced mammary tumour development by a high-fat diet. *Cancer Res.*, **44**, 2835–2840.

BERGER, M.R., MUSCHIOL, C., SCHMÄHL, D. & EIBL, H.J. (1987). New cytostatics with experimentally different toxic profiles. *Cancer Treat. Rev.*, **14**, 307–317.

BETH, M., BERGER, M.R., AKSOY, M. & SCHMÄHL, D. (1987). Comparison between the effects of dietary fat level and of calorie intake on methylnitrosourea-induced mammary carcinogenesis in female SD rats. *Int. J. Cancer*, **39**, 737–744.

CARROLL, K.K. & KHOR, H.T. (1971). Effects of level and type of dietary fat on incidence of mammary tumours induced in female Sprague-Dawley rats by 7,12-dimethylbenz(a)anthracene. *Lipids*, **6**, 415–420.

CAVE, W.T.Jr., DUNN, J.T. & MACLEOD, R.M. (1977). The effects of altered thyroid states on mammary tumour growth and pituitary gland function. *J. Natl Cancer Inst.*, **85**, 60–63.

CAVE, W.T.Jr. & HILF, R. (1987). Hormones and dietary lipids in breast cancer, 176–192. In *Growth Factors and Oncogenes in Breast Cancer*, Sluyser, M. (ed.) Ellis Horwood Series in Biomedicine, VCH Verlagsgesellschaft.

CHAN, P.C., HEAD, J.F., COHEN, L.A. & WYNDER, E.L. (1977). Influence of dietary fat on the induction of mammary tumours by N-nitrosomethylurea: associated hormone changes and differences between Sprague-Dawley and F344 rats. *J. Natl Cancer Inst.*, **59**, 1279–1283.

CLINTON, S.K., ALSTER, J.M., IMREY, P.B., SIMON, J. & VISEK, W.J. (1988). The combined effects of dietary protein and fat intake during the promotion phase of 7,12-dimethylbenz(a)anthracene-induced breast cancer in rats. *J. Nutr.*, **118**, 1557–1585.

COHEN, L.A. (1981). Mechanisms by which dietary fat may stimulate carcinogenesis in experimental animals. *Cancer Res.*, **41**, 3808–3810.

COHEN, L.A., CHOI, K.W. & WANG, C.X. (1988). Influence of dietary fat, calorie restriction, and voluntary exercise on N-nitrosomethylurea-induced mammary tumourigenesis in rats. *Cancer Res.*, **48**, 4276–4283.

DALY, J.M., REYNOLDS, H.M., ROWLANDS, B.J., DUDRICK, S.J. & COPELAND, E.M. (1980). Tumour growth in experimental animals. *Ann. Surg.*, **191**, 316–322.

DANGUY, A., LEGROS, N., DEVELEESCHOUWER, N., HEUSON-STENNON, J.A. & HEUSON, J.C. (1980). Effects of medroxyprogesterone acetate (MPA) on growth of DMBA-induced rat mammary tumours: histopathological and endocrine studies, 21–28. In *Role of Medroxyprogesterone in Endocrine-Related Tumours*, Iacobelli, S. & Di Marco, A. (eds), Raven Press: New York.

DONEGAN, W.L., HARTZ, A.J. & RIMM, A.A (1978). The association of body weight with recurrent cancer of the breast. *Cancer*, **41**, 1590–1594.

EISELE, R. (1986). Pathophysiologie des operativen Eingriffs, 67–78. In *Chirurgie*, Häring, R. & Zilch, H. (Hrsg.) Walter de Gruyter: Berlin, New York.

ENGELMAN, R.W., DAY, N.K., CHEN, R.F., TOMITA, Y., BAUER-SARDINA, I., DAO, M.L. & 1 other (1990). Calorie consumption level influences development of C3H/OU breast adenocarcinoma with indifference to calorie source. *Proc. Soc. Exp. Biol. Med.*, **193**, 23–30.

FELDMAN, J.M. & HILF, R. (1985). A role of estrogens and insulin binding in the dietary lipid alteration R3230AC mammary carcinoma growth in rats. *Cancer Res.*, **45**, 1964–1972.

GRAHAM, S., MARSHALL, J., METTLIN, C., RZEPKA, T., NEMOTO, T. & BYERS, T. (1982). Diet in the epidemiology of breast cancer. *Am. J. Epidemiol.*, **116**, 68–75.

GROSS, L. (1988). Inhibition of the development of tumours or leukemia in mice and rats after reduction of food intake. *Cancer*, **62**, 1463–1465.

HILF, R. (1982). Primary and permissive action of insulin in breast cancer, 123–137. In *Hormonal Regulation of Mammary Tumours. Peptide and Other Hormones*, Leung, B.S. (ed.), Eden Press: Montreal.

HILGARD, P., STEKAR, J., VOEGELI, R., ENGEL, J., SCHUMACHER, W., EIBL, H. & 2 others (1988). Characterization of the antitumour activity of hexadecylphosphocholine (D 18506). *Eur. J. Cancer Clin. Oncol.*, **24**, 1457–1461.

HIRAYAMA, T. (1978). Epidemiology of breast cancer with special reference to the role of diet. *Prev. Med.*, **7**, 173–195.

HIROHATA, T., NOMURA, A.M.Y., HANKIN, J.H., KOLONEL, L.N. & LEE, J. (1987). An epidemiologic study on the association between diet and breast cancer. *J. Natl Cancer Inst.*, **78**, 595–600.

HISLOP, T.G., COLDMAN, A.J., ELWOOD, J.M., BRAUER, G. & KAN, L. (1986). Childhood and recent eating patterns and risk of breast cancer. *Cancer Detect. Prev.*, **9**, 47–58.

HOLLANDER, M. & WOLFE, D.A. (1973). *Nonparametric Statistical Methods*. John Wiley: New York.

HOPKINS, G.J., KENNEDY, T.G. & CARROLL, K.K. (1981). Polyunsaturated fatty acids as promoters of mammary carcinogenesis induced in Sprague-Dawley rats by 7,12-Dimethylbenz(a)anthracene. *J. Natl Cancer Inst.*, **66**, 517–522.

HOWE, G.R., HIROHATA, T., HISLOP, G., ISCOVICH, J.M., YUAN, J.M., KATSOUYANNI, K. & 6 others (1990). Dietary factors and risk of breast cancer: combined analysis of 12 case-control studies (review). *J. Natl Cancer Inst.*, **82**, 561–569.

HULKA, B.S. (1989). Dietary fat and breast cancer: case-control and cohort studies. *Prev. Med.*, **18**, 180–193.

HURSTING, S.D., THORNQUIST, M. & HENDERSON, M.M. (1990). Types of dietary fat and the incidence of cancer at five sites. *Prev. Med.*, **19**, 242–253.

IP, C. & IP, M.M. (1980). Inhibition of mammary tumourigenesis by a reduction of fat intake after carcinogen treatment in young versus adult rats. *Cancer Lett.*, **11**, 35–42.

IP, C., IP, M.M. (1981). Serum estrogens and estrogen responsiveness in 7,12-dimethylbenz(a)anthracene-induced mammary tumours as influenced by dietary fat. *J. Natl Cancer Inst.*, **66**, 291–295.

IP, C., YIP, P. & BERNADIES, L.L. (1980). Role of prolactin in the promotion of dimethylbenz(a)anthracene-induced mammary tumours by dietary fat. *Cancer Res.*, **40**, 374–378.

JONES, D.Y., SCHATZKIN, A., GREEN, S.B., BLOCK, G., BRINTON, L.A., ZIEGLER, R.G. & 2 others (1987). Dietary fat and breast cancer in the National Health and Nutrition Examination Survey I Epidemiologic follow-up study. *J. Natl Cancer Inst.*, **79**, 465–471.

JOSE, D.G. & GOOD, R.A. (1973). Quantitative effects of nutritional protein and calorie deficiency upon immune responses to tumours in mice. *Cancer Res.*, **33**, 807–812.

KLURFELD, D.M., WELSCH, C.B., DAVIES, M.J. & KRITCHEVSKY, D. (1989). Determination of degree of energy restriction necessary to reduce DMBA-induced mammary tumourigenesis in rats during the promotion phase. *J. Nutr.*, **119**, 286–291.

KASANO, H., YASUTOMO, Y., KUGAI, N., NAGATA, N., INAGAKI, H., TANAKA, S. & 1 other (1990). Inhibition of estradiol uptake and transforming growth factor alpha secretion in human breast cancer cell line MCF-7 by an alkyl-lysophospholipid. *Cancer Res.*, **50**, 3172–3175.

KOZIOL, A.J. & DONNA, A.M. (1981). A distribution free test for tumour growth curve analyses with application to an animal tumour immunotherapy experiment. *Biometrics*, **37**, 383–390.

KRITCHEVSKY, D., WEBER, M.M., BUCK, C.L. & KLURFELD, D.M. (1986). Calories, fat and cancer. *Lipids*, **21**, 272–274.

KRITCHEVSKY, D., WELSH, C.B. & KLURFELD, D.M. (1989). Response of mammary tumours to calorie restriction for different time periods during the promotion phase. *Nutr. Cancer*, **12**, 259–269.

KUMAKI, T. & NOGUCHI, M. (1990). Effects of high dietary fat on the total DNA and receptor contents in rats with 7,12-dimethylbenz(a)anthracene-induced mammary carcinoma. *Oncology*, **47**, 352–358.

LASEKAN, J.B., CLAYTON, M.K., GENDRON-FITZPATRICK, A. & NEY, D.M. (1990). Dietary olive and safflower oils in promotion of DMBA-induced mammary tumourigenesis in rats. *Nutr. Cancer*, **13**, 153–163.

LUBIN, F., WAX, Y. & MODAN, B. (1986). Role of fat, animal protein, and dietary fiber in breast cancer etiology: a case-control study. *J. Natl Cancer Inst.*, **77**, 605–612.

MCCORMICK, D.L. (1989). Is the enhancement of rat mammary carcinogenesis by dietary fat a function of calorie intake. *Proc. Am. Ass. Cancer Res.*, **30**, A779.

MILLS, P.K., ANNEGERS, J.F. & PHILLIPS, R.I. (1988). Animal product consumption and subsequent fatal breast cancer risk among Seventh-Day Adventists. *Am. J. Epidemiol.*, **127**, 440–453.

NAKAMURA, Y., KODAMA, M. & KODAMA, T. (1981). Relation between adrenal function and 7,12-dimethylbenzanthracene-induced mammary cancer of the rat. *Gann.*, **72**, 679–683.

PHILLIPS, R.L. & SNOWDON, D.A. (1983). Association of meat and coffee use with cancers of the large bowel, breast, and prostate among Seventh-Day Adventists: Preliminary results. *Cancer Res. (Suppl)*, **43**, 2403s–2408s.

POLLARD, M., LUCKERT, H. & SYNDER, D. (1989). Prevention of prostate cancer and liver tumours in L-W rats by moderate dietary restriction. *Cancer*, **64**, 686–690.

ROE, F.J.C. (1981). Are nutritionists worried about the epidemic of tumours in laboratory animals? *Nutrition Soc. Proc.*, **40**, 57–65.

ROSEN, M., NYSTROM, L. & WALL, S. (1988). Diet and cancer mortality in the counties of Sweden. *Am. J. Epidemiol.*, **127**, 42–49.

SACHS, L. (1983). *Angewandte Statistik*. 6. Auflage, Springer Verlag: Berlin, Heidelberg, New York, Tokyo.

SANDOR, R.S. (1976). Effects of fasting on growth and glycolysis of the Ehrlich Ascites tumour. *J. Natl Cancer Inst.*, **56**, 427–428.

SAUER, L.A., NAGEL, W.O., DAUCHY, R.T., MICELI, L.A. & AUSTIN, J.E. (1986). Stimulation of tumour growth in adult rats *in vivo* during acute fast. *Cancer Res.*, **46**, 3469–3475.

SCHATZKIN, A., PIANTADOSI, S., MICCOZZI, M. & BARTEE, D. (1989). Alcohol consumption and breast cancer: a cross-national correlation study. *Int. J. Epidemiol.*, **18**, 28–31.

SHAO, R.P., DAO, M.L., DAY, N.K. & GOOD, R.A. (1990). Dietary manipulation of mammary tumour development in adult C3H/BI mice. *Proc. Soc. Exp. Biol. Med.*, **193**, 313–317.

SIEGEL, I., LIU, T.L., NEPOMUCENO, N. & GLEICHER, N. (1988). Effects of short-term dietary restriction on survival of mammary ascites tumour-bearing rats. *Cancer Invest.*, **6**, 677–680.

SUNDRAM, K., KHOR, H.T., ONG, A.S.H. & PATHMANATHAN, R. (1989). Effect of dietary palm oils on mammary carcinogenesis in female rats induced by 7,12-dimethylbenz(a)anthracene. *Cancer Res.*, **49**, 1447–1451.

VAN'T VEER, P., KOK, F.J., BRANTS, H.A.M., OCKHUIZEN, T., STURMANS, F. & HERMUS, R.J.J. (1990). Dietary fat and the risk of breast cancer. *Int. J. Epidemiol.*, **19**, 12–18.

VONDERHAAR, B.K. (1982). Effect of thyroid hormones on mammary tumour induction and growth, 138–154. In *Hormonal Regulation of Mammary Tumours. Peptide and other hormones.* Leung, B.S. (ed.) Eden Press: Montreal.

WELSCH, C.W. (1987). Enhancement of mammary tumourigenesis by dietary fat: review of potential mechanisms. *Am. J. Clin. Nutr.*, **45**, 192–202.

WELSCH, C.W., HOUSE, J.L., HERR, B.L., ELIASBERG, S.J. & WELSCH, M.A. (1990). Enhancement of mammary carcinogenesis by high levels of dietary fat: a phenomenon dependent on *ad libitum* feeding. *J. Natl Cancer Inst.*, **82**, 1615–1620.

WETSEL, W.C., ROGERS, A.E., RUTLEDGE, A. & LEAVITT, W.W. (1984). Absence of an effect of dietary corn oil content on plasma prolactin, progesterone, and 17-estradiol in female Sprague-Dawley rats. *Cancer Res.*, **44**, 1420–1425.

WILLET, W.C., STAMPFER, M.J., COLDITZ, G.A. & ROSNER, B.A. (1987). Dietary fat and the risk of breast cancer. *N. Eng. J. Med.*, **316**, 22–28.

WILLIAMS, C.M. & DICKERSON, J.W.T. (1987). Dietary fat, hormones and breast cancer: the cell membrane as a possible site of interaction of these two risk factors. *Eur. J. Surg. Oncol.*, **13**, 89–104.

YOSHIDA, H., FUKUNISHI, R., KATO, Y. & MATSUMOTO, K. (1980). Progesterone-stimulated growth of mammary carcinomas induced by 7,12-dimethylbenzanthracene in neonatally androgenized rats. *J. Natl Cancer Inst.*, **65**, 823–828.